# Altered White Matter Integrity at Illness Onset in Adolescents With a First Episode of Psychosis

**DOI:** 10.3389/fpsyt.2022.876793

**Published:** 2022-05-10

**Authors:** Soledad Romero, Elena de la Serna, Inmaculada Baeza, Isabel Valli, José Carlos Pariente, Marisol Picado, Nuria Bargalló, Gisela Sugranyes, Josefina Castro-Fornieles

**Affiliations:** ^1^Department of Child and Adolescent Psychiatry and Psychology, Institute of Neuroscience, Hospital Clinic Barcelona, Barcelona, Spain; ^2^Centro de Investigación Biomédica en Red de Salud Mental (CIBERSAM), Madrid, Spain; ^3^Institut d’Investigacions Biomediques August Pi i Sunyer (IDIBAPS), Barcelona, Spain; ^4^Department of Medicine, University of Barcelona, Barcelona, Spain; ^5^Image Diagnostic Center, Hospital Clinic of Barcelona, Barcelona, Spain

**Keywords:** diffusion, white matter (WM), early-onset psychosis, adolescence, neurodevelopment

## Abstract

**Background:**

Disruption in white matter integrity has been consistently observed in individuals with psychosis. However, whether such abnormalities are already present at illness onset or are related to downstream processes remains elusive. The study of adolescents with a recent onset of psychosis provides the opportunity to evaluate white matter integrity proximally to disease onset.

**Methods:**

Twenty-six adolescents (aged 15.9 ± 1.3 years) with a first episode of psychosis (FEP) (less than 6 months duration) were compared with 26 age and sex-matched healthy controls (HC) (16.8 ± 2 years). In participants with a FEP, clinical diagnoses were confirmed after a minimum of 1 year follow-up (main categories: schizophrenia, bipolar disorder, or schizoaffective disorder). Anatomical images and diffusion tensor sequences were acquired using a 1.5T scanner. Whole brain, voxel-wise group differences in fractional anisotropy (FA) were investigated between participants with a FEP and controls.

**Results:**

Relative to HC, FEP participants displayed decreased FA in the right posterior cingulate gyrus, encompassing the right superior and posterior corona radiata, and the right parahippocampal gyrus, including the cingulum and fornix. FEP patients showed no areas of increased FA relative to HC. The results remained significant after controlling for medication, cannabis use and intelligence.

**Conclusion:**

Our findings indicate that adolescents with recent onset of psychotic disorders show decreased white matter integrity in circuits implicated in cognitive functions and emotion regulation.

## Introduction

The dysconnectivity hypothesis suggests that psychotic illnesses arise not from regionally specific focal pathophysiology in the brain, but rather from abnormal or inefficient communication between brain regions and disturbances in the underlying pattern of white matter (WM) structural organization (1). Diffusion tensor imaging (DTI) provides an indirect measure of *in vivo* white matter integrity. Studies assessing DTI measures in subjects with schizophrenia (2) or bipolar disorders (3) in the ENIGMA consortium have identified widespread decrease in white matter microstructure in both conditions, with an overlap (4) in the corpus callosum and limbic structures. It has been posited that these brain abnormalities observed in patients with affective and non-affective psychosis may be due to alterations occurring during neurodevelopment. Neurodevelopmental models postulate that schizophrenia (and possibly bipolar disorder) is caused by environmental and/or genetic insults that occur during prenatal, perinatal, or early childhood periods, leading to alterations in brain structure and function and setting the stage for the clinical onset of psychosis (5). This *first hit* occurs during early developmental stages, setting the stage for further *hits*, which may occur during adolescence, at or near the onset of psychosis. This model was conceived to explain the age of onset of schizophrenia, as well as behavioral and cognitive abnormalities that can precede the emergence of psychotic symptoms (5–9).

However, whether changes in the brain observed in psychotic disorders are more linked to neurodevelopmental components of the disease or secondary to downstream illness processes still remains subject to debate (8–13). Studies assessing individuals with a first episode of psychosis (FEP) and short duration of the disease have the potential to help resolve some of these issues. In this line, a recent study has showed decreased whole-brain fractional anisotropy (FA) in medication-naive patients with a FEP (mean age 24 y/o) compared with controls. In FEP individuals, lower FA was correlated with longer duration of untreated psychosis and poorer subsequent response to antipsychotic treatment (14). In previous studies employing voxel-based analysis (VBA) in never-medicated subjects with a FEP, with a median duration of untreated psychosis of 6 months and mean age 28–30 y/o, a widespread decrease in FA was also reported, specifically, in the right superior longitudinal fasciculus (SLF), left fronto-occipital and inferior longitudinal fasciculus (ILF), right posterior limb of the internal capsula (PLIC), left anterior thalamic radiation and the corpus callosum (body, genu, and splenium) (15, 16). This suggests that in adults these abnormalities may be present since the beginning of the illness, and are not related with antipsychotic exposure.

Early-onset psychosis, which defines cases with an onset prior to age 18 (17), has been associated with poorer long-term outcomes compared to adult-onset psychosis (18, 19). This has been associated with the fact that emergence of the disease coincides with a critical period of neurodevelopment, both from a biological and psychosocial perspective (17). In child and adolescent onset schizophrenia, FA reductions have been reported in the left ILF, corticospinal tract, left optic radiation, corpus callosum, right anterior cingulum, left SLF and parietal and cerebellar white matter (20–25). These appear to follow a similar pattern to that observed in adult-onset patients in some (26), but not all studies (22). Only a small number of DTI studies have been undertaken in adolescents with bipolar disorder. These have reported reduced FA in orbito-frontal WM (27), limbic structures and corpus callosum (28–30). An important limitation of these studies is that the samples of early-onset schizophrenia and bipolar disorder are characterized by a long duration of illness (ranging from 6 to 30 months). Although such studies help to confirm that white matter abnormalities are present during adolescence, the fact that participants have been ill for a significant period of time makes it difficult to rule out whether these abnormalities already exist at illness onset and may therefore play a role in the primary pathophysiology of the disorder, or are a result of secondary illness processes (31).

Considering that no study so far has assessed white matter integrity in youth with a recent onset of psychosis, we set out to investigate this question in adolescents with a FEP and illness duration of less than 6 months. Based on previous studies, we hypothesized that adolescents with FEP would exhibit alterations in WM tracts with a distribution similar to that identified in adult populations (2–4) such as the anterior corona radiata, the limbic system, and the corpus callosum.

## Materials and Methods

### Participants

Twenty-six adolescents with first episode psychosis (FEP), aged 12–19 years [15.9 ± 1.3 years; male (M)/female (F) = 14/12] were recruited during their first admission to the inpatient unit at the Department of Child and Adolescent Psychiatry and Psychology of the Hospital Clinic of Barcelona, Spain. Adolescents presented with positive psychotic symptoms such as delusions and/or hallucinations of less than 6 months duration. Lifetime and current psychopathology (DSM-IV criteria) was ascertained by a child and adolescent psychiatrist with expertise in psychosis (I.B), at intake and after a one-year follow-up period.

Twenty-six healthy control (HC) adolescents (16.8 ± 2 years M/F = 17/9) were recruited from the same geographic area. All HC were free of current or past DSM—IV axis I diagnoses and 1st degree family history of psychosis. HC were interviewed using the Spanish version of the Schedule for Affective Disorders and Schizophrenia for School-Age Children Present and Lifetime Version (K-SADS-PL) (32).

Exclusion criteria for all participants of the study included intellectual disability defined as an intelligence quotient (IQ) < 70 with impaired functioning, major systemic medical illness, serious head injury, pregnancy, and imaging counterindications. An additional exclusion criterion for subjects with a FEP was the presence of a comorbid axis I disorder at the time of evaluation which could account for psychotic symptoms, such as autism spectrum disorders, post-traumatic stress disorder, or acute stress disorder. Substance use was not considered an exclusion criterion if positive symptoms persisted for more than 2 weeks after a negative urine test.

The study was approved by the local Ethical Review Board. All patients and their parents or legal guardians provided written informed consent.

### Clinical Measures

Psychotic symptoms were rated using the Positive and Negative Syndrome Scale (PANSS) (33), and mood symptoms were assessed with the Hamilton Rating Scale for Depression (34) and the Young Mania Rating Scale (35). Symptom severity was additionally quantified with the Clinical Global Impression (CGI).

Intelligence quotient was estimated using the Block Design and Vocabulary subtest of the Wechsler Adult Intelligence Scale—III Revised (WAIS III) or the Wechsler Intelligence Scale for Children—Revised (WISC-R), depending on the subject’s age.

### Image Acquisition

Images were acquired on a 1.5 Tesla General Electric GENESIS SIGNA scanner. A 3D structural MRI was acquired using a T1-weighted FSPRG sequence (acquisition plane: axial, TR: 12 ms, TE: 5.17 ms, TI: 300 ms, NEX: 1, flip angle: 9°, FOV: 256 × 256, *n*°slices: 108, slice thickness: 1.5 mm, voxel size: 0.98 × 0.98 × 1.5 mm^3^) and a DTI sequence using EPI pulse sequence with diffusion-weighting (acquisition plane: axial, TR: 10000 ms, TE: 79.9 ms, Flip Angle: 90°, FOV: 256 × 256, *n*°slices: 24, slice thickness: 5 mm, b: 1000 s/mm, *n*° of diffusion gradients: 25, voxel size: 1 × 1 × 5 mm^3^).

### Image Processing

All T1-weighted images (T1w) were manually reoriented to stereotactic space, according to the anterior-posterior commissure plane, using SPM12 (Statistical Parametric Mapping, Welcome Department, University College of London, United Kingdom^1^).

Group tissue probability maps (TPM) representative of our sample were created from the T1w structural images, using the Segment tool of SPM12. The individual TPMs (including gray matter– GM-, white matter– WM-, cerebrospinal fluid, bone, soft tissue, and air/background) were spatially normalized to Montreal Neurological Institute (MNI) space, meaned and smoothed with a 12-mm isotropic Gaussian Kernel. The customized TPMs were used instead of the SPM12 default adult TPMs to improve the segmentation of the structural images.

Diffusion-weighted images (DWI) were corrected for eddy current distortions and motion using eddy correct tool in FSL (FMRIB Software Library, Oxford, United Kingdom^2^) (36). FA maps were calculated from the corrected DWI using FDT^3^ and co-registered to the T1w images using a two-step registration (FLIRT, FMRIB’s Linear Image Registration Tool and FNIRT, FMRIB’s Non-linear Image Registration Tool). T2-weighted images were used to correct the geometric image distortions which are inherent to the echo-planar imaging (EPI) readout (37, 38).

Inter-subject registration was performed using DARTEL (39). GM and WM were employed to calculate the individual flow fields used for the transformation of the co-registered FA maps to MNI space. Finally, the normalized FA maps were smoothed with an 8 mm FWHM Gaussian kernel.

### Group Analysis

The threshold masking value used for establishing which voxels should undergo statistical analysis was absolute and set at 0.2. This threshold, defined to exclude voxels unlikely to belong to white matter, was set by visual inspection of the processed images and the resulting mask. The analysis included a 2-sample *t*-test to examine differences in FA between HC and FEP patients, where age and gender were used as nuisance variables to control for potential confounder effects. The statistical criteria were as follows: all voxels surviving a statistical threshold of *p* < 0.001 (uncorrected) were considered for analysis, but only clusters surviving a threshold of *p* < 0.05 FWE (family-wise error) corrected for multiple comparisons are reported.

General linear model (GLM) models were fitted to those clusters which showed between group differences in FA. MANCOVA analyses were employed to examine the influence of the cumulative doses of antipsychotic drugs, and to compare individuals with, versus those without, a history of substance use and lithium treatment. Given that the groups differed in global IQ, the between group analyses were also repeated covarying for IQ.

The anatomical localization of each cluster showing significant between-group differences in FA was determined with the tool Atlasquery of FSL,^4^ using all anatomical templates (Harvard-Oxford cortical and subcortical structural atlases, Jülich histological atlas, JHU DTI-based white-matter atlases, Oxford thalamic connectivity atlas, Talairach atlas, and MNI structural atlas).

Exploratory analyses examining the association between symptoms and DTI measures and divided by subgroups and cannabis use are included as Supplementary Material.

## Results

### Demographic and Clinical Characteristics

Clinical and socio-demographic characteristics of the sample are described in Table 1. Duration of psychotic symptoms at the time of the scan ranged from 12 to 170 days with a mean of 56 days (SD = 45). All subjects in the FEP group were receiving treatment with second generation antipsychotics (risperidone: *n* = 25; mean daily dose: 4.8 ± 2 mg; olanzapine: *n* = 1; daily dose: 15 mg); mean duration of treatment at the time of the scan was 28 ± 31 days. Additionally, eight patients also received treatment with lithium (mean duration of treatment: 32 ± 40 days), and six with an antidepressant (mean duration of treatment: 22 ± 19 days). Ten FEP subjects and no HC reported a history of cannabis use. Diagnoses at intake and after one year follow-up, and clinical measurements for FEP patients are reported in Table 1. At one year follow-up, approximately 40% of adolescents met criteria for bipolar disorder, 40% for schizophrenia, and 20% for schizoaffective disorder.

**TABLE 1:** Demographics and clinical characteristics of the sample.

*Demographics*	FEP *n* = 26	HC *n* = 26	Stat	*p* value
Age (mean, SD)	15.9 ± 1.3	16.8 ± 2	*t* = –1.9	0.06
Sex (%, male)	14 (54%)	17 (65%)	χ ^2^ = 0.7	0.4
***Clinical characteristics***				
Duration of psychosis, days (mean, SD)	56 ± 46	N/A		
Cumulative dose of antipsychotic*	11329 ± 18028	N/A		
Estimated IQ	81 ± 15	106 ± 13	*t* = 5.9	<0.001
Cannabis abuse	10 (39%)	N/A		
**Diagnosis at intake (*n*, %)**				
Psychotic disorder NOS	13 (50%)	N/A		
Bipolar disorder type I	9 (35%)			
Schizophrenia	2 (8%)			
Major depressive episode with psychotic features	2 (8%)			
**Diagnosis after 1 year follow up (*n*, %)**				
Bipolar disorder type I	10 (39%)	N/A		
Schizophrenia	11 (42%)			
Schizoaffective disorder	5 (19%)			
**Scales (mean, SD)**				
PANSS total	67 ± 8.5	N/A		
Hamilton Depression Rating Scale	12.3 ± 6.5			
Young Mania Rating Scale	15.4 ± 10.6			
Clinical Global Impression	6.3 ± 0.7			

*Chlorpromazine equivalent × number of days receiving antipsychotic medication.

### Voxel-Based Analysis of Diffusion Tensor Imaging

**FIGURE 1:**
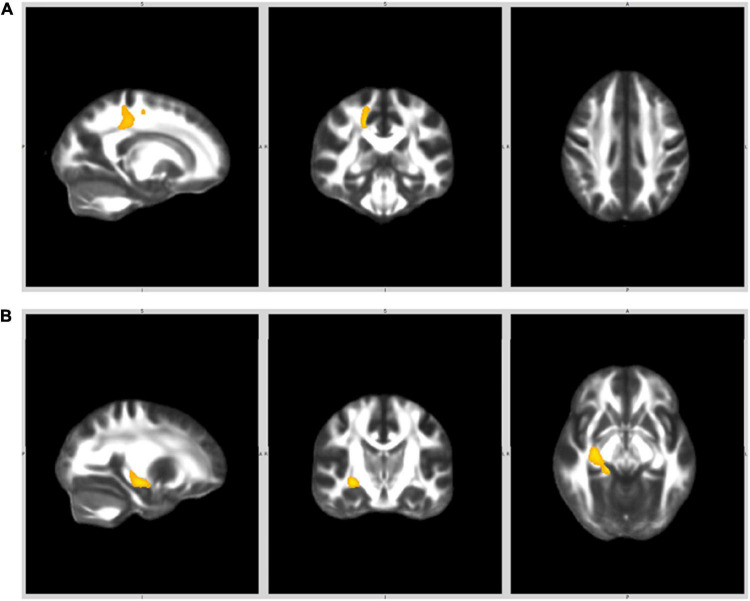
White matter clusters where FA was significantly lower in FEP compared to HC. **(A)** Right posterior cingulate gyrus (right superior and posterior corona radiata). **(B)** Right parahippocampal gyrus (cingulam, fornix).

First episode of psychosis patients showed decreased FA in two different clusters compared with HC (Table 2). These clusters correspond to (1) the right posterior cingulate gyrus encompassing the right superior and posterior corona radiata and (2) the right parahippocampal gyrus encompassing the cingulum and fornix. FEP patients showed no areas of increased FA relative to controls (Figure 1). Results remained significant after controlling for medication exposure (cumulative doses of antipsychotic drugs, and lithium treatment), cannabis use and IQ. In exploratory analyses we found no differences in FA between diagnostic subgroups or according to lithium treatment or cannabis use (presented in Supplementary Material).

**TABLE 2:** Areas with decreased FA values in FEP vs. HC in the whole-brain analysis at a cluster level.

Brain region	WM tract	Laterality	Peak MNI coordinate	Cluster size	Tmax	*p* value (FWE-corrected)
Posterior cingulate gyrus	Posterior Corona Radiata	Right	17, –37, 37	1685	4.3	0.05
	Superior corona radiata					
Parahippocampal gyrus	Cingulum (hippocampus)	Right	30, –17, –8	2512	4.6	0.01
	Fornix					

## Discussion

To our knowledge this is the first study to employ DTI, using a voxel-based whole brain analysis approach, in adolescents with very recent onset psychoses. In our sample, adolescents with a FEP showed decreased FA values relative to HC in the right posterior cingulate gyrus, encompassing the right superior and posterior corona radiata, and the right parahippocampal gyrus, including the cingulum and fornix. FEP patients displayed no areas of increased FA in comparison with HC.

The corona radiata consists of projection fibers that interconnect the cerebral cortex with the thalamus and brainstem in both directions *via* the internal capsule. The internal capsule/corona radiata is the anatomic linkage that supports cognitive and perceptual and motor systems in the cortex. The parahippocampus receives widespread input from the cortex and is a primary afferent of the hippocampus (40). The cingulum bundle is a white matter fiber tract that underlies the cingulate cortex, which courses longitudinally over the corpus callosum (41). All connections entering and exiting the cingulate cortex pass through this bundle, which spans the subrostral areas of the frontal lobe beneath the corpus callosum and reaches the parahippocampal gyrus and uncus in the medial temporal lobe. It plays a key role in regulating emotional processing and social cognition (42), functions which have been found to be impaired in individuals with psychotic disorders (41). The fornix connects the hippocampus with the septal region and the mammillary nucleus of the hypothalamus (40), and has been implicated in cognitive disturbances and memory function in schizophrenia (43).

The current results are partially in line with those reported by the ENIGMA consortium that identified widespread significant reductions in FA in schizophrenia patients (*n* = 1963), with the largest effects in the anterior corona radiata and the corpus callosum (2), as well as in bipolar subjects (*n* = 1482), in whom the largest effect size was observed within the corpus callosum and the cingulum (3). These results have been replicated recently by the COCORO consortium, which found that patients with schizophrenia (*n* = 696) and bipolar disorder (*n* = 211) share similar white matter microstructural differences in the body of the corpus callosum and in the limbic system, encompassing the fornix and cingulum (4), as compared to HC.

Our findings replicate changes in FA in tracts connecting limbic structures, observed in adult patients with established disease. However, we did not find changes in the corpus callosum or other long range association tracts. A recent multi-site diffusion MRI study (44) investigating differences in FA across the lifespan (14–65 years) between subjects with schizophrenia (*n* = 600) and HC (*n* = 493) reported three distinct patterns of white matter disruption: limbic connections appeared selectively vulnerable to early developmental anomalies, whereas long-range intra-hemispheric association tracts displayed shorter maturational windows and faster declines, consistent with accelerated aging processes in schizophrenia (44). In contrast, in the same study changes in callosal-fibers, which were present from the onset of the illness, became more pronounced with increasing age. Based on these findings, the authors suggested that white matter changes in schizophrenia may be sensitive to age and illness stage, and differ in a tract-specific manner (44). Our results are in line with these findings, as our sample of adolescents with a FEP exhibited disruptions in white matter integrity in limbic fibers, which has been associated with early developmental disruption, but not in long-range association fibers or callosal fibers that may be more prominent in later stages of the illness, in agreement with the progressive brain deterioration observed in patients with chronic schizophrenia.

Taken together, our results suggest that findings of decreased FA in connections between limbic structures (right posterior cingulate gyrus and right parahippocampal gyrus) may be present since the onset of psychosis (40, 44). This is supported by the fact that some of these abnormalities have also been documented in healthy young siblings of psychotic patients (45), and in clinical high-risk samples (46), which suggests that such white matter abnormalities may be related to neurodevelopmental components present proximally to the occurrence of psychosis, and are unlikely to be solely the result of secondary illness processes. Importantly, the affected areas are relevant to the symptoms of the disorder, as they have been postulated to underpin some of the cognitive and emotional symptoms characteristic of these patients (10, 11, 47).

Several methodological considerations need to be taken into account when interpreting the present findings. First, the sample comprised adolescents with a FEP, in whom differential diagnosis was formulated at follow-up. Therefore, the observed alterations in white matter tracts may be characteristic of the acute psychotic phase and may not be generalizable to stabilized schizophrenia or bipolar disorder patients (48, 49). In addition, the onset of psychotic symptoms was defined as the moment when positive symptoms emerged, and not based on impaired cognitive or social functioning. Second, the fact that all affective patients displayed psychotic symptoms at onset may limit the comparability with other, less severe, non-psychotic adult and pediatric bipolar samples. Third, all FEP youth were taking medication at the time of scanning, since the severity of the symptoms required that subjects were treated promptly, and scanning was performed once patients were clinically stabilized. Nevertheless, the duration of antipsychotic treatment was relatively short (mean = 28 days) and after controlling for medication status the results remained unchanged. Although largely unknown, psychotropic medications have been postulated to exert either no effect early on in the treatment or to have an ameliorative effect on white matter integrity (50–52). Fourth, the small sample size may have limited the capacity to detect between group differences, especially when examining diagnostic subgroups, hence why these analyses are presented as exploratory. While we acknowledge that other DTI measures (such as mean or axial diffusivity) may also have been informative, the limited power also led us to minimize comparisons and focus on fractional anisotropy.

In sum, our results suggest that disruption in white matter integrity is present during adolescence and from the onset of clinical illness, affecting limbic associative and projection fibers. These findings may help future studies seeking to understand the diagnostic specificity and prognostic potential of white matter changes in adolescents with FEP.

## Data Availability Statement

The raw data supporting the conclusions of this article will be made available by the authors, without undue reservation.

## Ethics Statement

The studies involving human participants were reviewed and approved by Comitè Ètic d’Investigació Clínica, Hospital Clinic Barcelona. Written informed consent to participate in this study was provided by the participants’ legal guardian/next of kin.

## Author Contributions

SR, ES, IB, and GS contributed to the design, acquisition, analysis and interpretation of data for the work. JP and MP contributed to the acquisition, analysis and interpretation of data for the work. IV, NB, and JC-F contributed to the analysis and interpretation of data for the work. SR and GS drafted the first version of the manuscript and all the authors revised it critically for important intellectual content. All authors provided approval for publication of the content and agreed to be accountable for all aspects of the work in ensuring that questions related to the accuracy or integrity of any part of the work were appropriately investigated and resolved.

## Conflict of Interest

The authors declare that the research was conducted in the absence of any commercial or financial relationships that could be construed as a potential conflict of interest.

## Publisher’s Note

All claims expressed in this article are solely those of the authors and do not necessarily represent those of their affiliated organizations, or those of the publisher, the editors and the reviewers. Any product that may be evaluated in this article, or claim that may be made by its manufacturer, is not guaranteed or endorsed by the publisher.

## Footnotes

1
http://www.fil.ion.ucl.ac.uk/spm

2
http://www.fmrib.ox.ac.uk/fsl

3
http://fsl.fmrib.ox.ac.uk/fsl/fslwiki/FDT

4
https://fsl.fmrib.ox.ac.uk/fsl/fslwiki/Atlasquery
