# Supplementary material for: Altered White Matter Integrity at Illness Onset in Adolescents With a First Episode of Psychosis
Source: Front Psychiatry. 2022 May 10;13:876793. doi: 10.3389/fpsyt.2022.876793 (PMC9127302; doi:10.3389/fpsyt.2022.876793)
Supplement: Supplementary file 1 [file Data_Sheet_1.PDF]

Supplemental material

1. Relationship between changes in FA and clinical variables (symptom severity, antipsychotic medication dose and lithium treatment).

We used a general linear model to assess the relationship between FA values in the right posterior cingulate and scores in the Positive And Negative Symptom Scale (PANSS) total scale (F=0.007, p=0.934), and the Clinical Global Impression (CGI) scale (F=0.06, p=0.939), and the dose of antipsychotic medication transformed into chlorpromazine equivalents (F=3.630, p=0.07). Similar results were found when studying the relationship between FA values in the right parahippocampal region and the PANSS total (F=0.515, p=0.481) and CGI (F=2.046, p=0.167) scores, and antipsychotic medication doses transformed into chlorpromazine equivalents (F=0.049, p=0.827).
Using ANOVA, we examined whether there were differences in FA values in the group of patients with first episode psychosis (FEP) according to whether they were receiving treatment with lithium (n=8 receiving lithium vs n=18 without). We did not find differences in the FA value in either the posterior cingulate (0.315 ± 0.027 vs. 0.322 ± 0.026, F=0.41, p=0.53) or in the parahippocampal regions (0.271± 0.007 vs. 0.277± 0.024, F=0.385, p=0.541).

2. FA group differences on the basis of cannabis use and FEP sub-diagnosis at follow up.

Using ANOVA, we examined possible group differences in DTI measures on the basis of cannabis use and FEP diagnoses at follow-up.

When comparing FEP patients according to cannabis use (n=10 with versus n=16 without) we did not find any differences in the FA value in either the right posterior cingulate (0.323 ± 0.029 vs. 0.318 ± 0.023, F=0.273, p=0.6) or right parahippocampal region (0.269± 0.0143 vs. 0.278± 0.023, F=1.419, p=0.245).

When comparing FEP patients according to diagnoses at follow-up (adolescents with schizophrenia spectrum disorder, n=16 vs adolescents with bipolar disorder, n=10), no significant differences were found in the FA value in either the right posterior cingulate (0.321 ± 0.027 vs. 0.319 ± 0.025, F=0.028, p=0.87) or parahippocampal regions (0.276± 0.025 vs. 0.272 ± 0.008, F=0.242, p=0.627).
